# Supplementary material for: Multiple molecular events underlie stochastic switching between 2 heritable cell states in fungi
Source: PLoS Biol. 2022 May 20;20(5):e3001657. doi: 10.1371/journal.pbio.3001657 (PMC9162332; doi:10.1371/journal.pbio.3001657)
Supplement: S1 File — A PDF file containing Supporting information figures (A–J) and table (A). Each figure is followed by a legend and data underlying the figures can be found at (https://osf.io/6e9vz/). Table A. Switching frequencies. Switching frequencies as calculated by counting sectors on colonies grown on solid agar plates. Data come from 3 independent experiments containing multiple plates per strain. Numbers of colonies and sectors are pooled across plates. The percent of colonies with at least 1 sector is calculated. Standard error is the standard error of a sample proportion (√ [p (1-p) / n)]). Fig A. Opaque cell growth in microfluidic traps. Two examples of microfluidic traps with growing opaque cells. Strain contains Wor1-GFP fusion protein. Fig B. Cell length distributions. (A) Cell lengths of pairs of switching cells (mothers versus their daughters), including switching cells containing Wor1-mGFP. Typical white and opaque cell distributions are shown on margins. (B) Cell length distributions (violin plots) of different cell types. For the Wor1-GFP strain, data come from 3 independent experiments that were pooled in main text Fig 2A. Fig C. Nucleus loss during mitosis in switching cells. (A) Representative example of a cell division where the mother cell nucleus enters the elongated daughter cell but returns to the mother cell. Strain contains Wor1-GFP fusion protein. (B) Representative example of cell divisions where the mother cell nucleus enters the elongated daughter cell and remains in the daughter cell, creating a polyploid cell. There are 2 cell divisions with this pattern in this example. Strain contains Wor1-GFP fusion protein. Fig D. Semiautomated image analysis pipeline. (A) Representative original images of 2 time points for 1 field. (B) Same images as (A) after adjustment for (x,y) movement (note black regions at image edge) and automated segmentation of cells (magenta colored lines defining cell borders). (C) Same images as (A) and (B) with overlay of manually [file pbio.3001657.s001.pdf]

Table A

White to Opaque switching					
Genotype	Plates	Colonies	Sectors	Percent Switch	Fold Change
WT	11 (3,3,5)	1340	17	1.27 (+/- 0.306)	-
Wor1 motif deletion	11 (3,3,5)	1790	8	0.447 (+/- 0.158)	2.83 down
Wor1-GFP	11 (3,3,5)	948	491	51.8 (+/- 1.62)	40.8 up
Wor1-mGFP	11 (3,3,5)	1603	31	1.93 (+/- 0.344)	1.52 up

Opaque to White switching					
Genotype	Plates	Colonies	Sectors	Percent Switch	Fold Change
WT	11 (3,3,5)	1926	87	4.52 (+/- 0.473)	-
Wor1 motif deletion	11 (3,3,5)	1159	224	19.3 (+/- 1.16)	4.28 up
Wor1-GFP	11 (3,3,5)	1633	0	<0.06	>75 down
Wor1-mGFP	11 (3,3,5)	1707	52	3.05 (+/- 0.42)	1.48 down

Table A – Switching frequencies
Switching frequencies as calculated by counting sectors on colonies grown on solid agar plates. Data comes from three independent experiments containing multiple plates per strain. Numbers of colonies and sectors are pooled across plates. The percent of colonies with at least one sector is calculated. Standard error is the standard error of a sample proportion ($\sqrt{[p (1-p) / n]}$).

Figure A

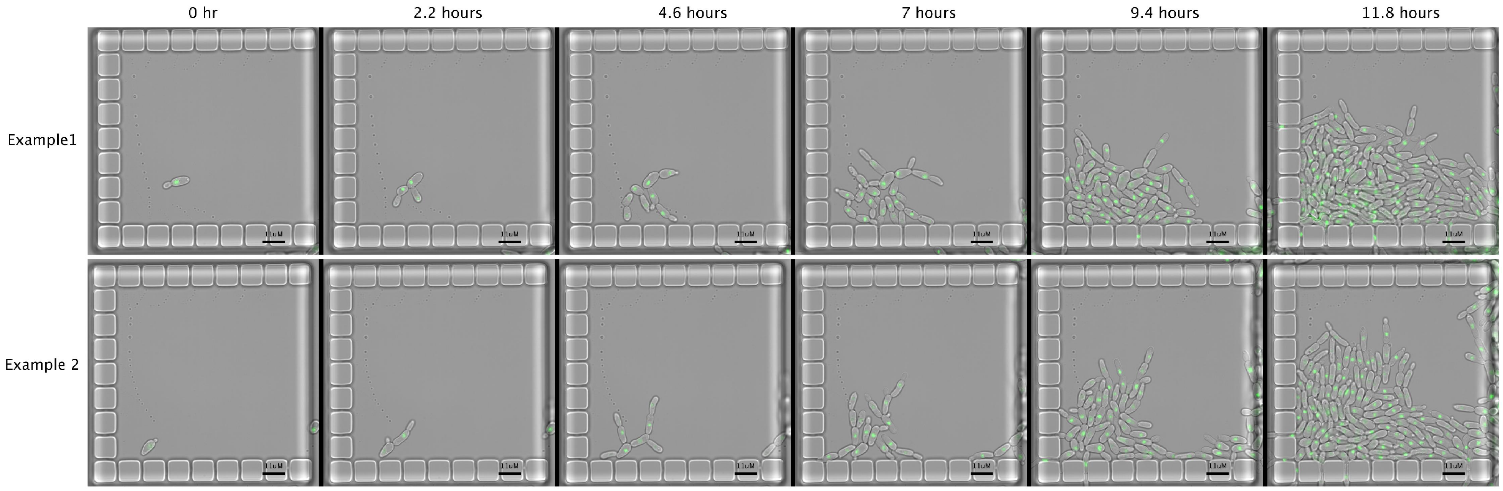

Figure A – Opaque cell growth in microfluidic traps
Two examples of microfluidic traps with growing opaque cells. Strain contains Wor1-GFP fusion protein.

Figure B

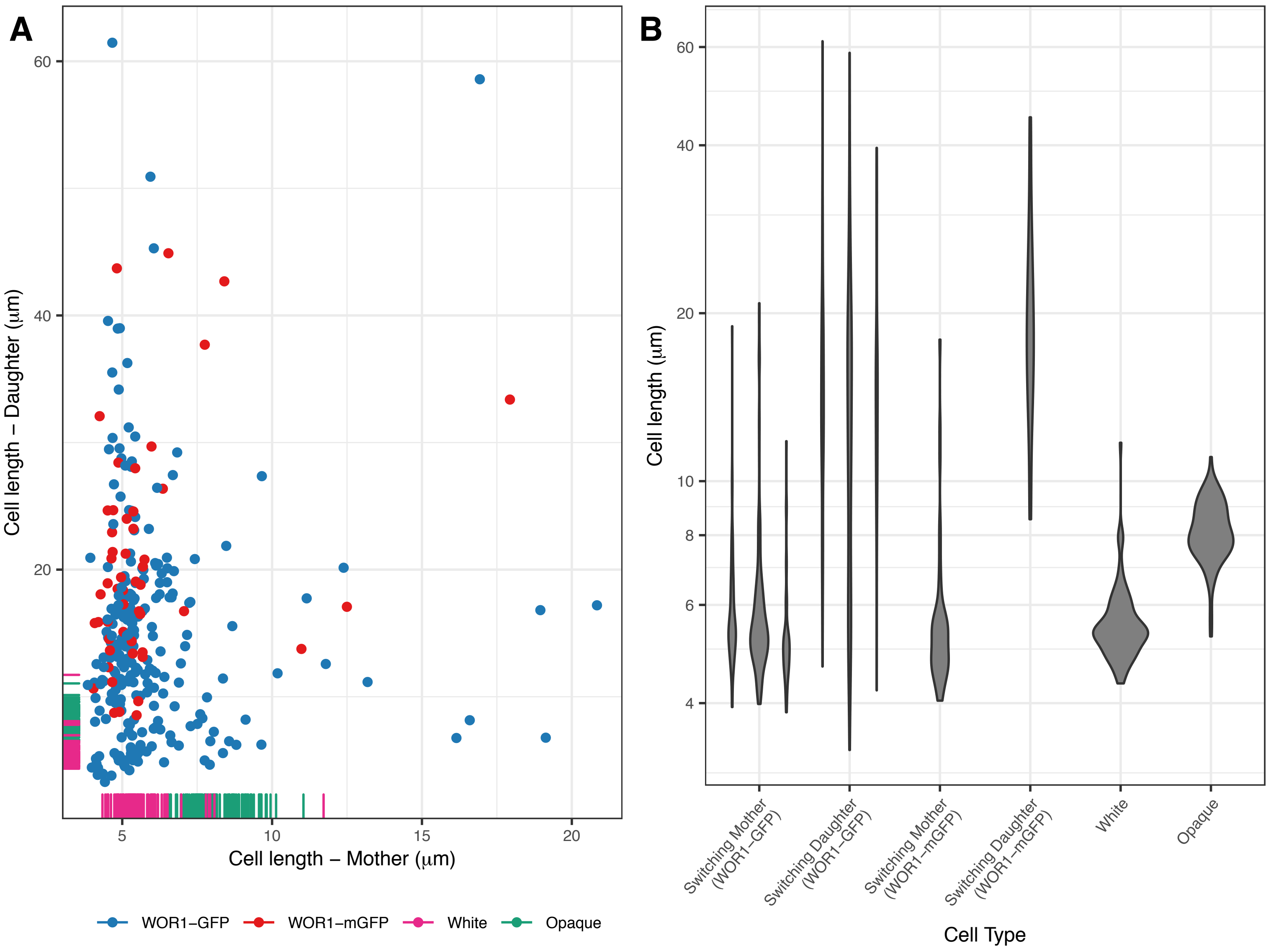

Figure B– Cell length distributions
A) Cell lengths of pairs of switching cells (mothers versus their daughters), including switching cells containing Wor1-mGFP. Typical white and opaque cell distributions are shown on margins.
B) Cell length distributions (violin plots) of different cell types. For the Wor1-GFP strain, data comes from three independent experiments that were pooled in main text Figure 2A.

Figure C

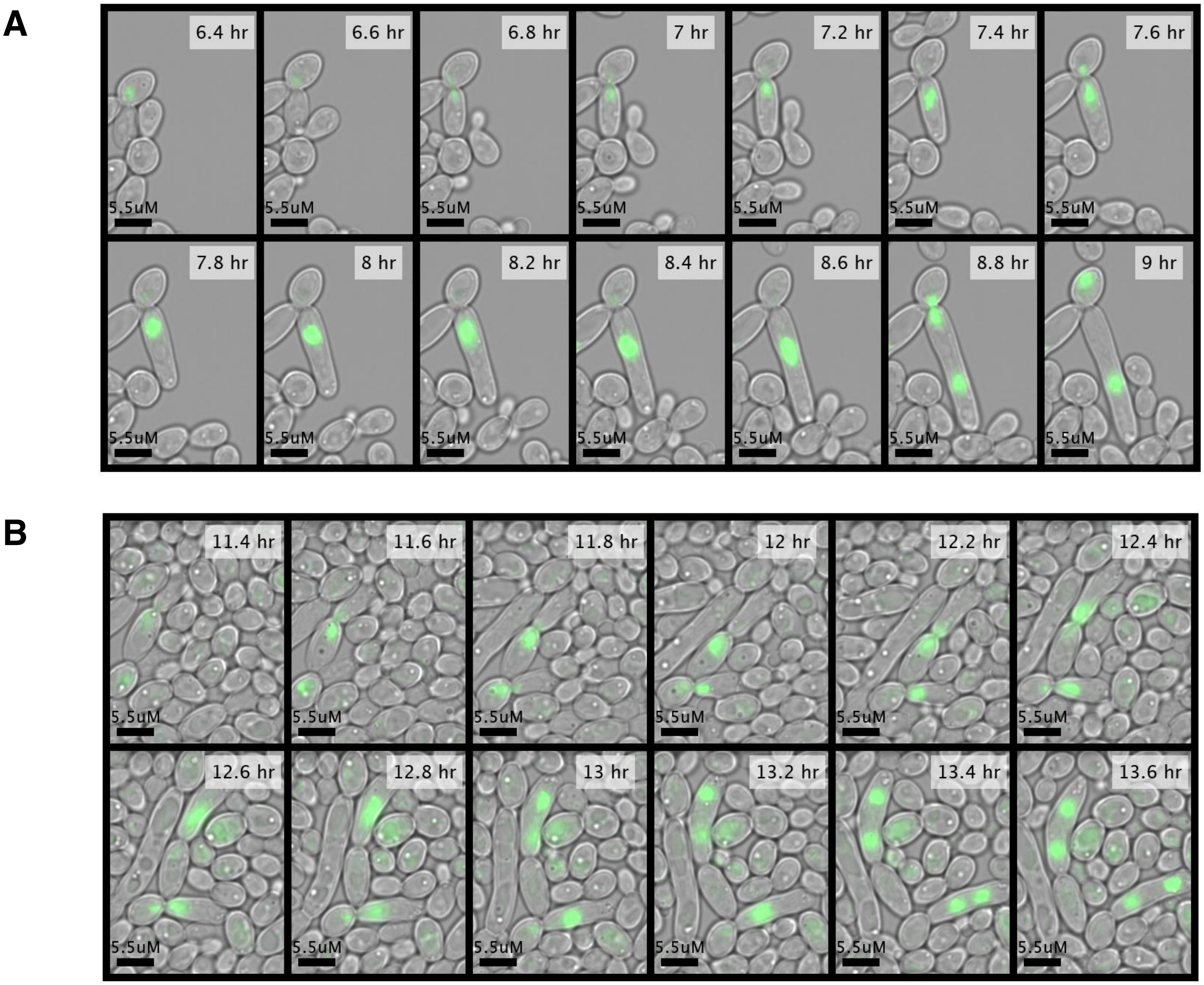

Figure C – Nucleus loss during mitosis in switching cells
A) Representative example of a cell division where the mother cell nucleus enters the elongated daughter cell but returns to the mother cell. Strain contains Wor1-GFP fusion protein.
B) Representative example of cell divisions where the mother cell nucleus enters the elongated daughter cell and remains in the daughter cell, creating a polyploid cell. There are two cell divisions with this pattern in this example. Strain contains Wor1-GFP fusion protein.

Figure D

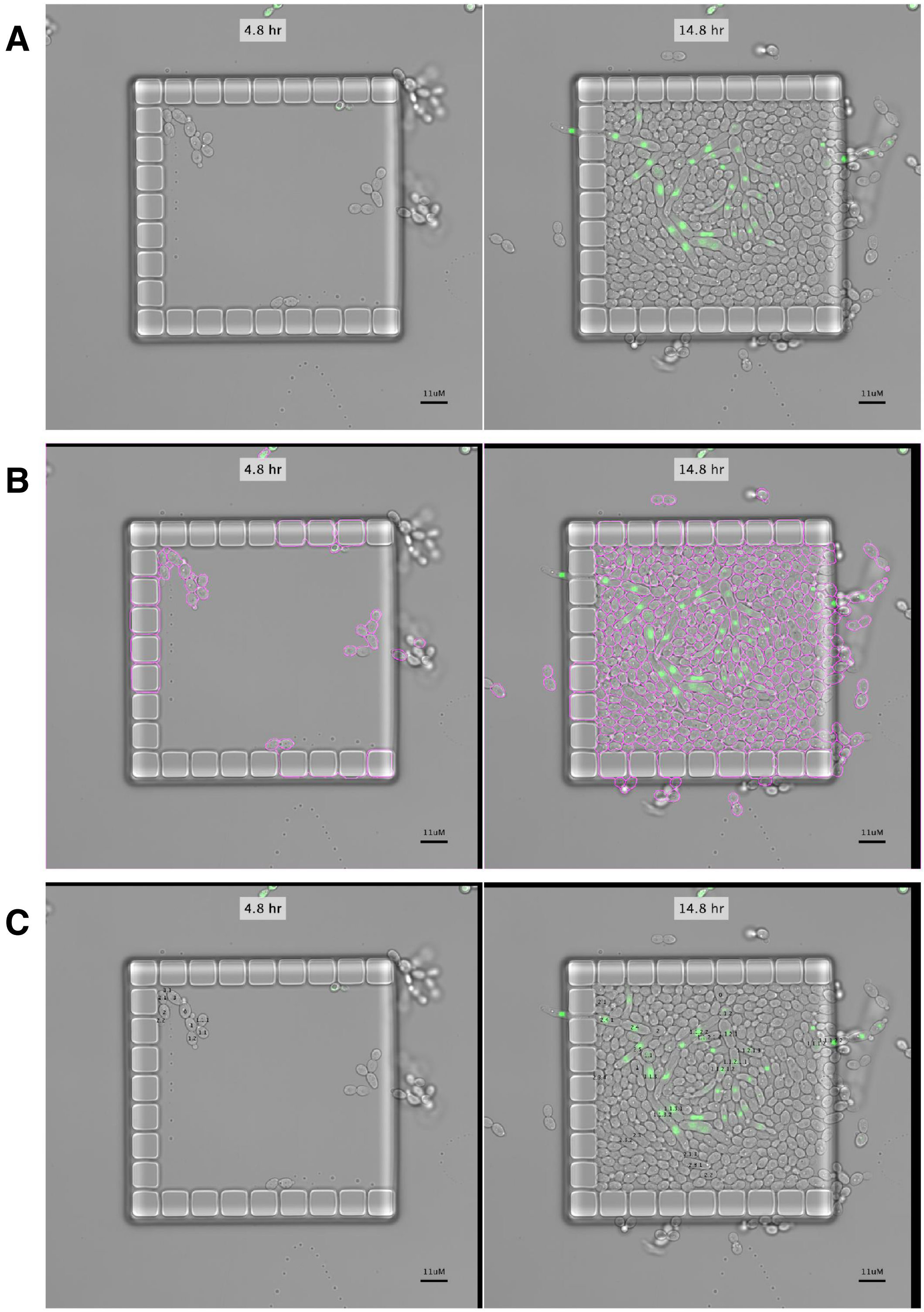

Figure D – Semi-automated image analysis pipeline
A) Representative original images of two time-points for one field.
B) Same images as (A) after adjustment for (x,y) movement (note black regions at image edge) and automated segmentation of cells (magenta colored lines defining cell borders).
C) Same images as (A) and (B) with overlay of manually defined cell identities, pedigree information is explicitly encoded in cell names (e.g., cell “2.1” is the first daughter of cell “2” which in turn is the second daughter of cell “0”).

Figure E

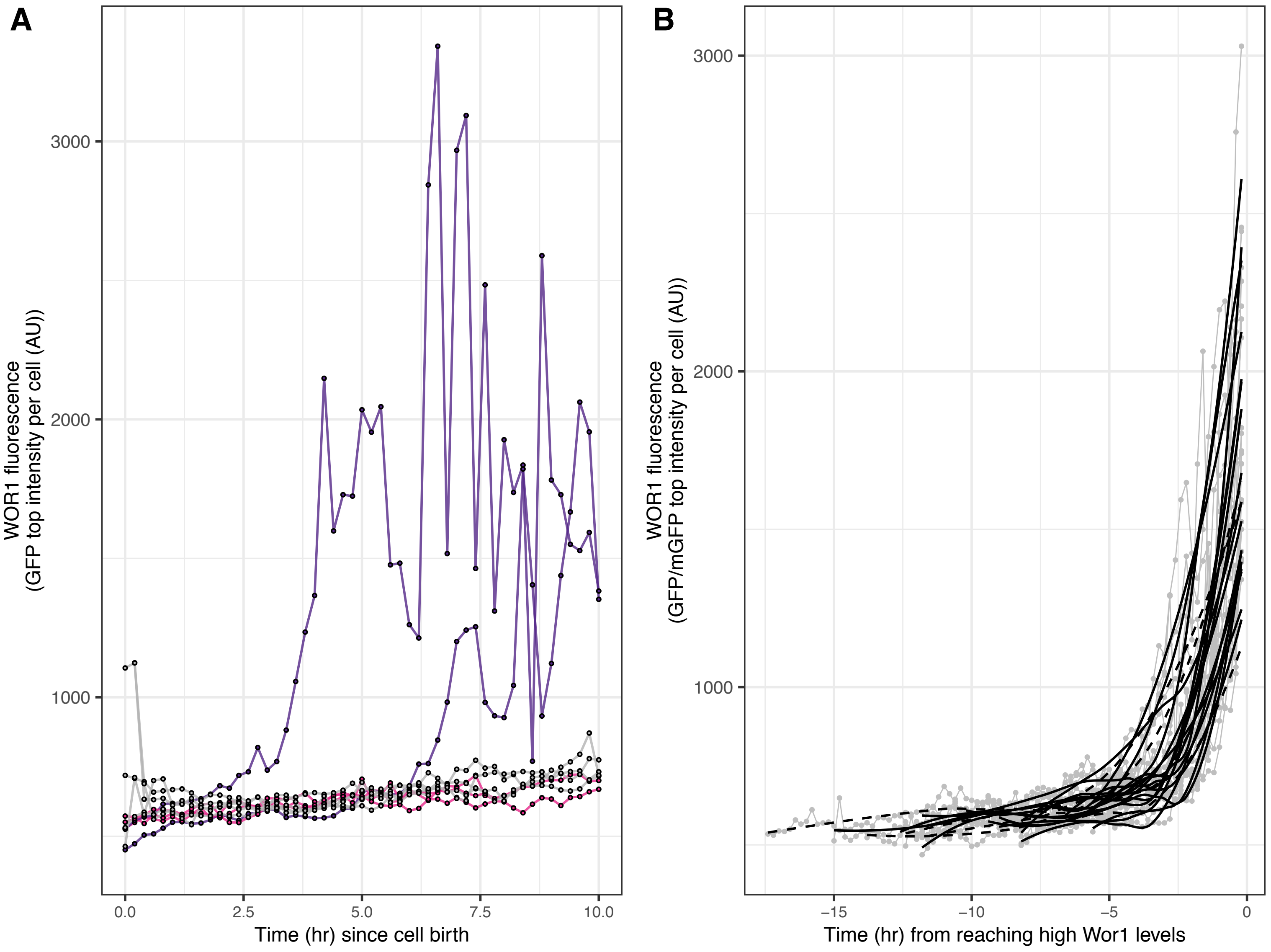

Figure E – Fluorescence (GFP/mGFP) levels in single cells.
A) Additional Wor1 expression level traces in single cells. Traces are of white cells that do not contain Wor1-GFP (grey) and white cells that do contain Wor1-GFP (pink/purple). X-axis represents time from cell birth. Cells that are not actively switching have similar fluorescence levels to typical white cells that do not contain GFP.
B) The same examples of Wor1 activation in single switching cells shown in main text Figure 2D, lined up by the approximate time cells reach high Wor1 levels. Although the time between the birth of a switching cell and its switching varies, reaching high levels of Wor1 from background fluorescence levels takes approximately 3 hours.

Figure F

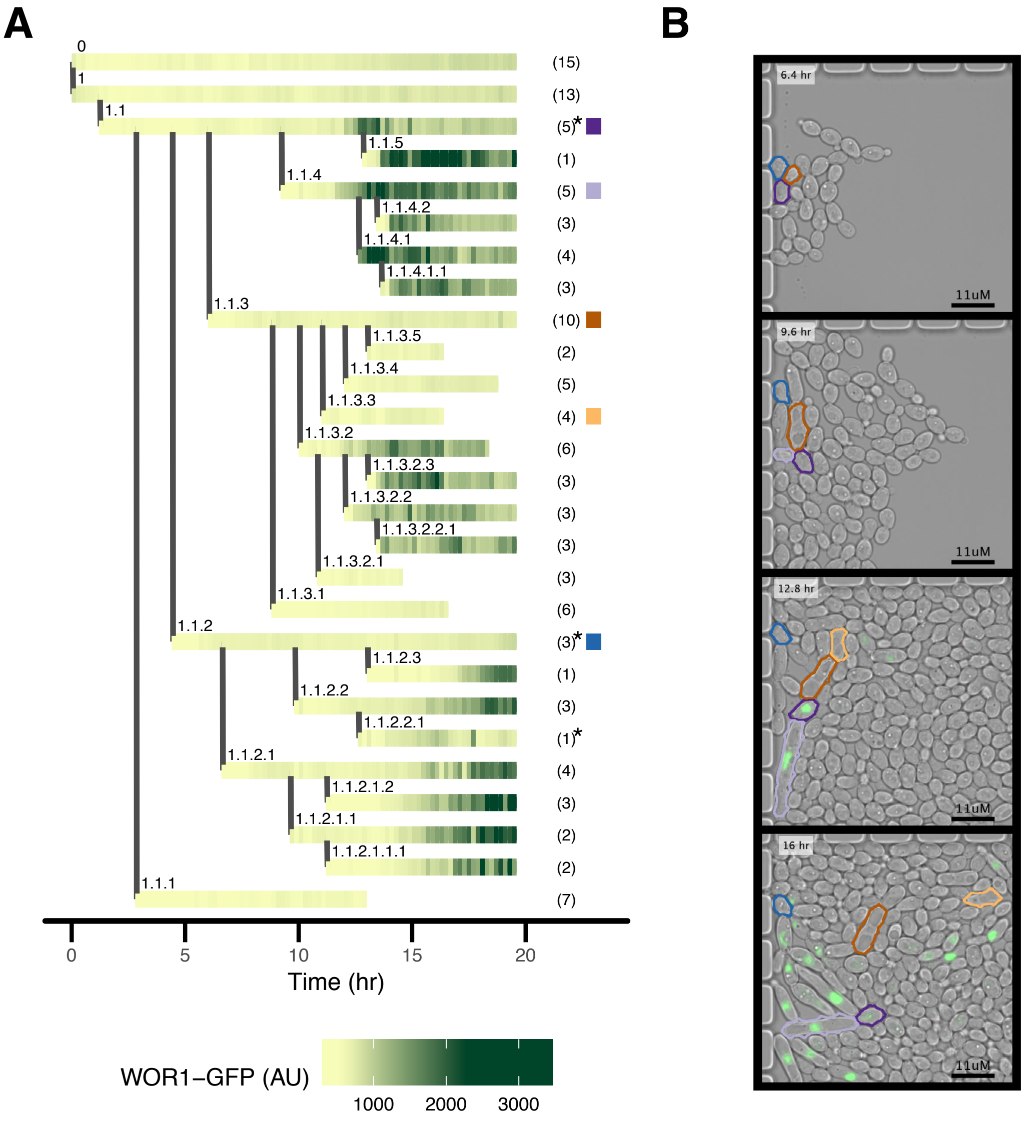

Figure F – Mixed fate pedigree example, Wor1-GFP
A) Representative pedigree in which multiple groups of cells activated Wor1. Horizontal lines represent single cells; vertical lines represent budding of a daughter cell. Every horizontal line is made up of small tiles colored by Wor1-GFP fluorescence. Numbers within parentheses on the right of the pedigree represent the number of budded daughter cells per cell within the time period shown; not all daughters are depicted in the pedigree. An asterisk represents that the cell lost its nucleus to its daughter cell. Colored square tiles single out particular cells that are depicted in (B).
B) Subset of images representing data shown in (A). Particular cells are outlined in colors corresponding to (A). Cell outlines are based on automated image analysis.

Figure G

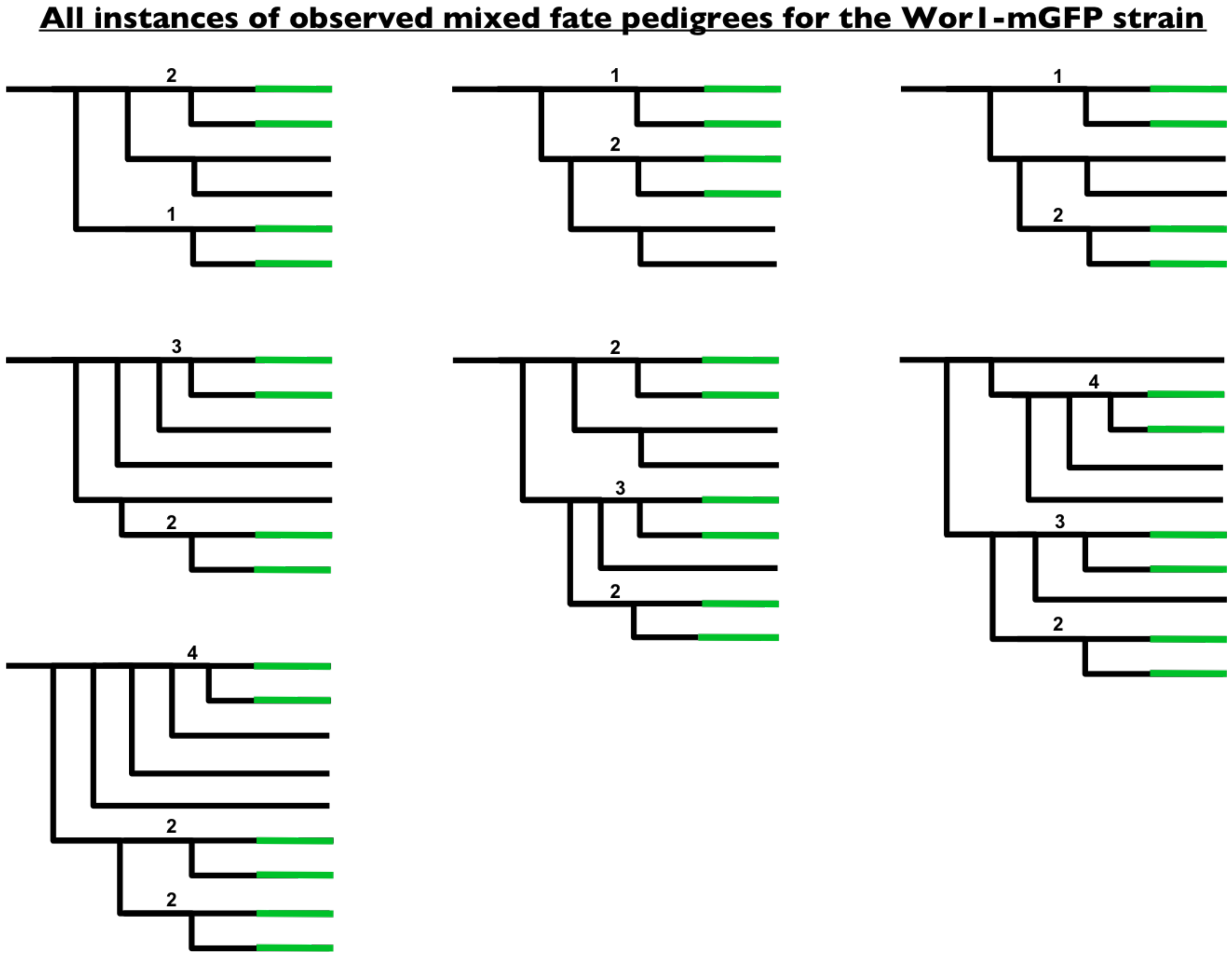

Figure G – Mixed fate pedigrees
Schematics of all mixed fate pedigrees observed for the Wor1-mGFP strain. Numbers represent the number of cell divisions separating switching cells from the establishment of the predisposed pedigree. Representation is for illustrating cell relationships and do not accurately reflect cell division times or timing/ intensity of Wor1 activation.

Figure H

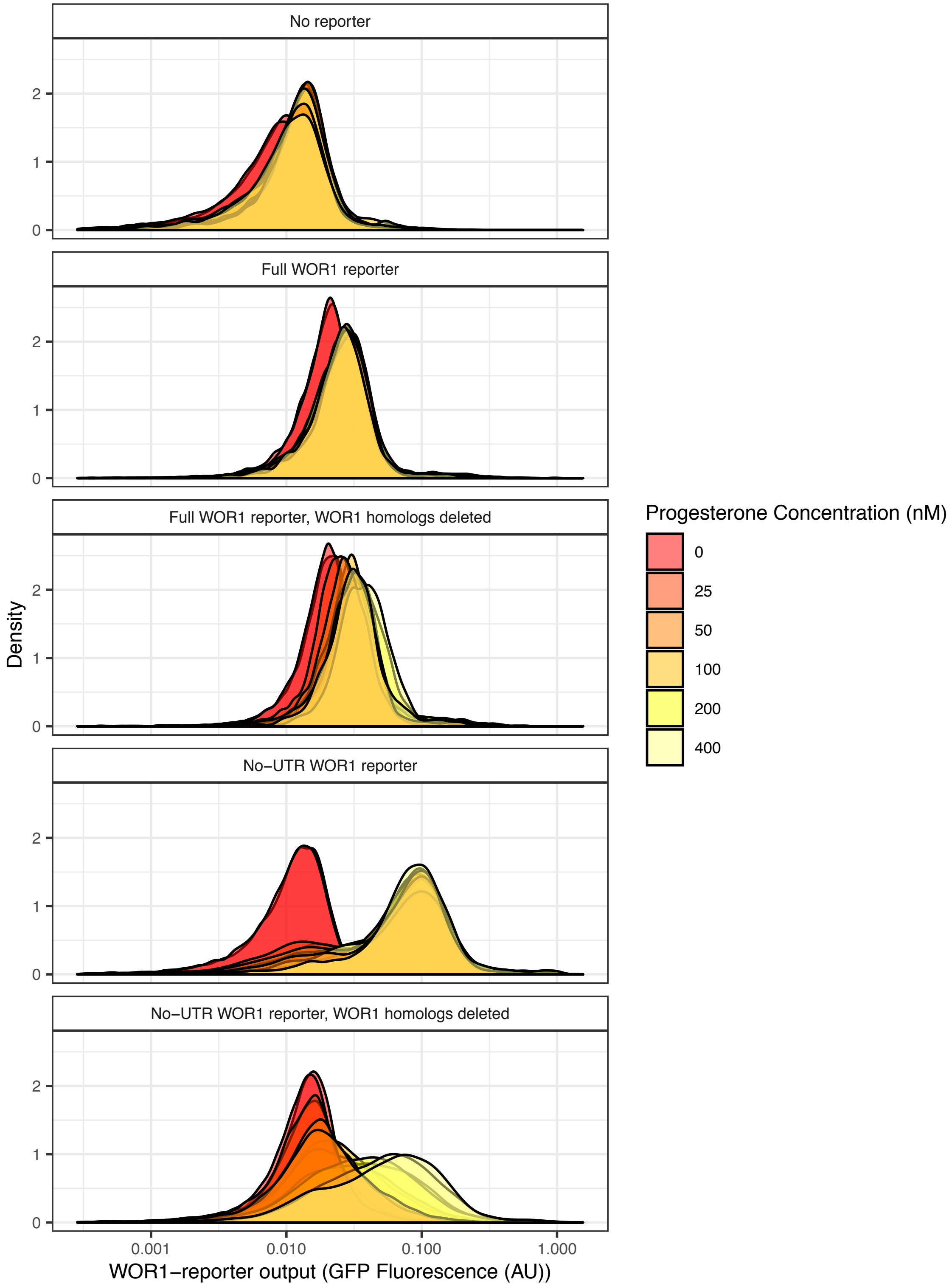

Figure H - Alternative Wor1 transcriptional reporters in S. cerevisiae
Distributions of Wor1 transcriptional reporters; all strains shown are inducing Wor1 protein with increasing progesterone concentrations and were independently constructed from the strains shown in the main text. “Full” reporter contains both the Wor1 7KB control region and promoter and the Wor1 2KB 5’UTR. The “No-UTR” reporter contains the Wor1 control region and the Cyc1 core promoter and 5’UTR as explained in the methods section.

Figure I

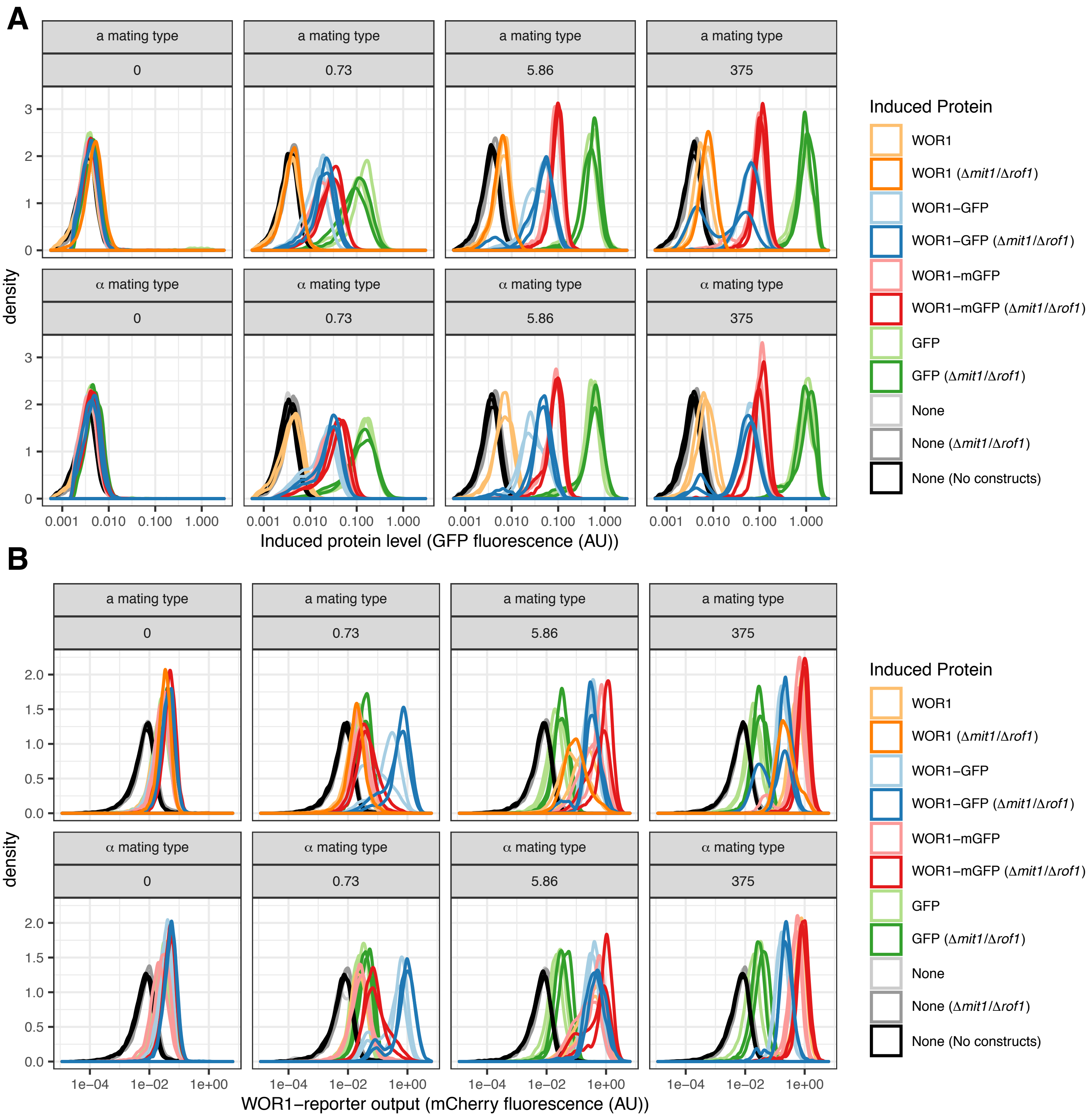

Figure I - Distributions of Wor1 protein and reporter levels
Distributions of GFP (A) and mCherry (B) fluorescence; data is shown for all strains in a subset of 4 hormone concentrations (nM progesterone). Note bimodal distributions of GFP fluorescence when inducing Wor1-GFP at high hormone concentrations in (A). Note low-level expression of the Wor1 transcriptional reporter compared to auto-fluorescence in (B).

Figure J

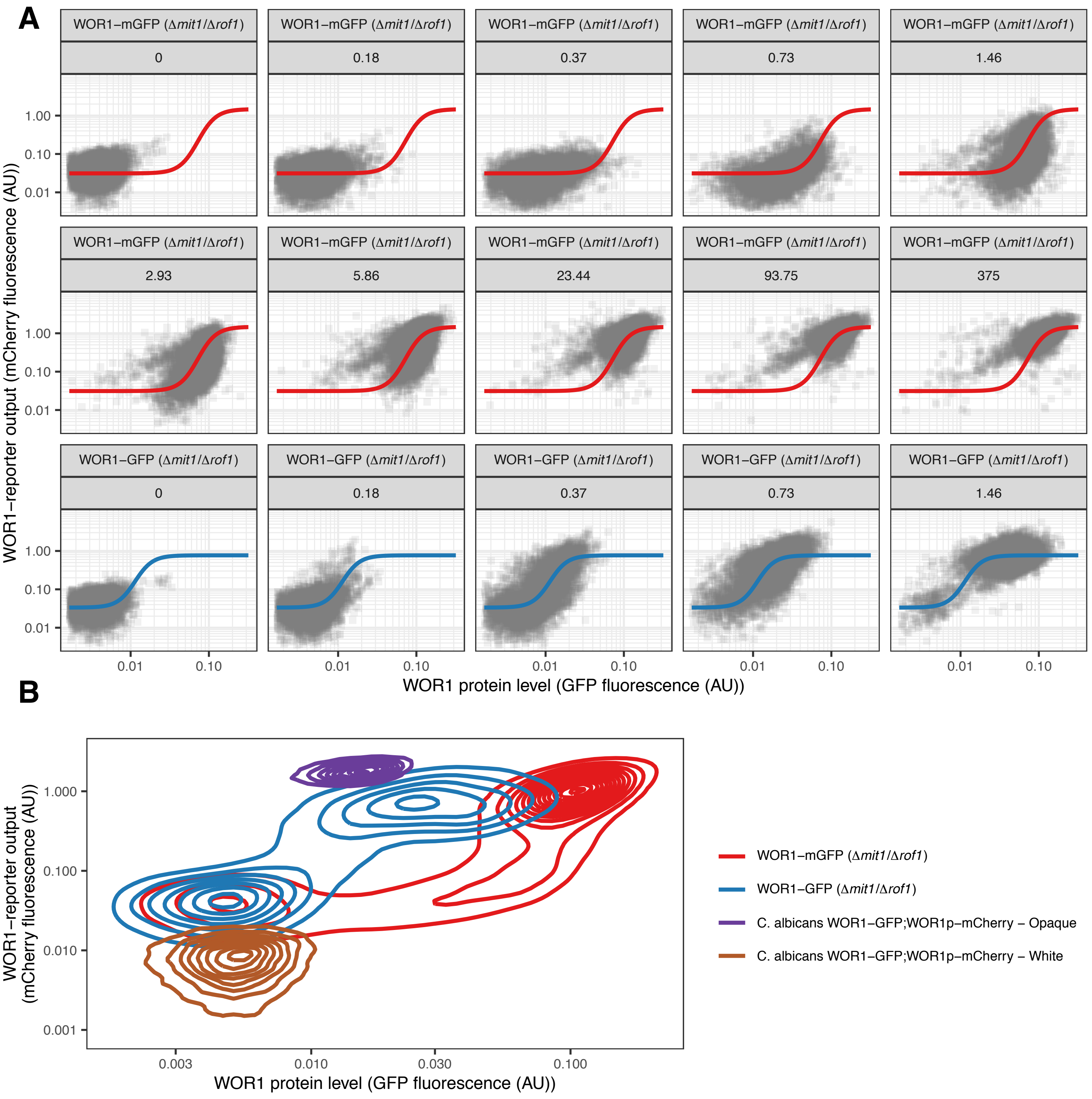

Figure J – Distributions of Wor1 protein and reporter levels
A) Same data as main text Figure 4C, separated by hormone concentration (nM progesterone).
B) 2D density plots of flow cytometry data. S. cerevisiae Wor1-GFP and Wor1-mGFP data (same as A) are shown alongside data for white and opaque C. albicans cells containing a Wor1-GFP fusion protein and a transcriptional reporter encompassing the 7KB control region of Wor1 followed by mCherry (no 5'UTR sequence).
