# Supplementary material for: Multiple molecular events underlie stochastic switching between 2 heritable cell states in fungi
Source: PLoS Biol. 2022 May 20;20(5):e3001657. doi: 10.1371/journal.pbio.3001657 (PMC9162332; doi:10.1371/journal.pbio.3001657)
Supplement: Attachment — Submitted filename: PBIOLOGY-D-21-03197 Johnson Candida.docx [file pbio.3001657.s004.docx]

Stochastic fate switching is the basis for the formation of non-genetic phenotypic heterogeneity. While multiple instances of such phenotypic switching are observed across several model systems, the molecular basis still remains elusive. In this paper, Ziv et al used the white to opaque switching phenotype in *Candida* albicans to study the mechanism that leads to such heterogeneity by focusing on the inheritance and positive autoregulation of Wor1 expression across generations. High expression of Wor1 is associated with the formation of opaque cells. The paper reports and tries to link to distinct phenomena: the pattern of switching in *Candida*  cells and the ability of the product of a transplanted Wor1 gene to activate its own transcription in *Saccharomyces cerevisiae*. The *cerevisiae* experiment reveals very strong positive feedback and it is possible that both the same positive feedback exists in *Candida* but given the large phylogenetic distance between the organisms this is a hypothesis more than either an inference or a conclusion and the writing of the paper should clearly reflect this distinction even though this is a *Discovery* article.

The paper reports that there appears to be an event that occurs before the detectable increase in Wor1 levels and which increases the probability that Wor1 will rise and cells will become opaque. The most interesting observation is that in a lineage where this event has occurred, some cells can activate Wor1 and others cannot but that all the cells that activate Wor1 do so synchronously. The authors argue that there must be two events to describe this behavior, but these could be closely related. For example, the first event could be inducing Wor1 expression to a level below their threshold of detection that leads to a slow, but essentially deterministic increase in Wor1 concentration that will eventually lead to strong Wor1 expression and a commitment to the opaque state. To explain the failure of some cells in a lineage to make this switch, you need only appeal to the stochastic expression of some inhibitor that interrupts this positive feedback loop.

There are two principal weaknesses to the paper. The first is whether the key event that leads to opaque cells exceeding a certain level of Wor1 expression, something else, or Wor1 expression plus something else. Testing necessity would be hard but the observation that Wor1 dimerization, through GFP, increases switching supports the notion that increased Wor1 expression stimulates switching. The second is the large gap between the *Candida* and *Saccharomyces* experiments. The behavior in *Saccharomyces* is suggestive but not much more and it’s unclear how much measuring a Hill coefficient in a different organisms amplifies previous work that demonstrated positive autoregulation of Wor1 expression. Whether these weaknesses preclude publication as a *Discovery* article is an editorial decision.

Major recommendations:

1. An interesting question would be to see if the current data can put a limit on how many generations the state that leads to later synchronous Wor1 activation can exist for. To put it the other way, how distantly related can two cells in a lineage be that both participate in synchronous Wor1 activation.

2. A convenient way to confirm bistability arising due to the positive autoregulation of Wor1 in *Saccharomyces* would be to use the flow cytometry data used for Figure 5B. If one induces Wor1-GFP to a level that is around the half-maximal threshold, the expression level of mCherry driven from the Wor1 promoter at later generations should be heterogeneous i.e. some cells in the population should show strong mCherry expression and other should have none. An important control would be to show all cells have mCherry OFF in low Wor1-GFP expressed strain and all cells have mCherry ON in high Wor1-GFP expressed strain. This is an easy experiment to do with the strains reported in the paper

Minor comments:

1. In Figure 1D, the authors can put Wor1-GFP in one panel and Wor1-mGFP in the other rather than two examples to show the differences in switching rate between the two constructs.

2. Line 235, Page 215: Is the formation of polyploid cells associated with pseudohyphae formation. It might be interesting to some readers if the authors observe something similar.

3. Line 264, Page 216: The authors have mentioned here ‘they were always inherited across subsequent cell divisions’ but later they show that some of the cells in the lineage don’t turn on Wor1. The authors should revise this statement to avoid inconsistency.

4. Line 252, Page 216: It is not exactly clear from the figure 3A-C if the time taken to each maximum level for Wor1 is 3 hours. In Figure 3C, the mother cells take ~6 hours to reach maximum expression levels. Therefore, it would be optimal to put a distribution of switching time and present the mean/standard deviation.

5. In the figure 3B, the data can be plotted starting from the cell division rather than imaging time to show that they more or less take the same amount of time to reach activation thresholds. The mean and standard deviation of the time can be put on the graph. Additionally, the plots corresponding to mGFP and GFP need to be colored separately.
