# Supplementary material for: Multiple molecular events underlie stochastic switching between 2 heritable cell states in fungi
Source: PLoS Biol. 2022 May 20;20(5):e3001657. doi: 10.1371/journal.pbio.3001657 (PMC9162332; doi:10.1371/journal.pbio.3001657)
Supplement: Attachment — Submitted filename: Ziv_Response_to_Reviewers.docx [file pbio.3001657.s005.docx]

We thank the reviewers for their comments on our manuscript. Please find our **response (in bold)** to reviewer comments (in roman) and *changes to the text (in italics)* below.

Editorial requests:

IMPORTANT: We considered your paper as a Discovery Report. The maximum allowable number of Figs for this article type is 4, but you currently have 5. Please either combine some of these Figs or move some material to the supplement.

**We have combined figures 2 and 3.**

Please note that as a condition of publication PLOS' data policy requires that you make available all data used to draw the conclusions arrived at in your manuscript. If you have not already done so, you must include any data used in your manuscript either in appropriate repositories, within the body of the manuscript, or as supporting information (N.B. this includes any numerical values that were used to generate graphs, histograms etc.).

**We have deposited all data and scripts on Open Science Framework (OSF),**

**DOI: 10.17605/OSF.IO/6E9VZ (https://osf.io/6e9vz/)**

Reviewer #1:

Stochastic fate switching is the basis for the formation of non-genetic phenotypic heterogeneity. While multiple instances of such phenotypic switching are observed across several model systems, the molecular basis still remains elusive. In this paper, Ziv et al used the white to opaque switching phenotype in Candida albicans to study the mechanism that leads to such heterogeneity by focusing on the inheritance and positive autoregulation of Wor1 expression across generations. High expression of Wor1 is associated with the formation of opaque cells. The paper reports and tries to link to distinct phenomena: the pattern of switching in Candida cells and the ability of the product of a transplanted Wor1 gene to activate its own transcription in Saccharomyces cerevisiae. The cerevisiae experiment reveals very strong positive feedback and it is possible that both the same positive feedback exists in Candida but given the large phylogenetic distance between the organisms this is a hypothesis more than either an inference or a conclusion and the writing of the paper should clearly reflect this distinction even though this is a Discovery article.

**We thank the reviewer for assessing our article. We believe the experiments in *Saccharomyces cerevisiae* are valuable for quantifying the parameters of Wor1 autoregulation in a way that would be impossible in the native system. We do not think (and reviewer 2 also mentions in the cross-comments) that the phylogenetic distance between the species is a problem but we now clarify in the discussion that other regulators may change these parameters in *Candida albicans*.**

*We show here that Wor1 regulation of its own promoter is ultrasensitive even in the absence of other C. albicans regulators. While our measurements provide a baseline for the autoregulation of Wor1, other factors in C. albicans may change these parameters.*

The paper reports that there appears to be an event that occurs before the detectable increase in Wor1 levels and which increases the probability that Wor1 will rise and cells will become opaque. The most interesting observation is that in a lineage where this event has occurred, some cells can activate Wor1 and others cannot but that all the cells that activate Wor1 do so synchronously. The authors argue that there must be two events to describe this behavior, but these could be closely related. For example, the first event could be inducing Wor1 expression to a level below their threshold of detection that leads to a slow, but essentially deterministic increase in Wor1 concentration that will eventually lead to strong Wor1 expression and a commitment to the opaque state. To explain the failure of some cells in a lineage to make this switch, you need only appeal to the stochastic expression of some inhibitor that interrupts this positive feedback loop.

**We have added the suggestion that stochastic expression of an inhibitor could explain the appearance of mixed fate pedigrees to the discussion.**

*According to this hypothesis, the first stochastic event during white-opaque switching might be a rare burst of low-level Wor1 transcription. This state would be heritable over only a small number of cell divisions, defining the small pedigrees of cells predisposed to switch.* *The second stochastic event would then create variation in the amount of Wor1 between cells, perhaps because of variation in translation efficiency, uneven partitioning during division or the stochastic expression of an inhibitor. Only certain cells in the pedigree would accumulate sufficient Wor1 protein to excite the Wor1 positive feedback loop, leading to high level Wor1 expression and subsequent cell-type switching.*

There are two principal weaknesses to the paper. The first is whether the key event that leads to opaque cells exceeding a certain level of Wor1 expression, something else, or Wor1 expression plus something else. Testing necessity would be hard but the observation that Wor1 dimerization, through GFP, increases switching supports the notion that increased Wor1 expression stimulates switching. The second is the large gap between the Candida and Saccharomyces experiments. The behavior in Saccharomyces is suggestive but not much more and it’s unclear how much measuring a Hill coefficient in a different organisms amplifies previous work that demonstrated positive autoregulation of Wor1 expression. Whether these weaknesses preclude publication as a Discovery article is an editorial decision.

**We believe the experiments described in this paper have laid the foundation for analyzing white to opaque switching at a level not possible before. Our initial observation of small mixed fate lineages allows us to start dissecting the events that lead to switching, including what determines which lineages are predisposed to switch and what determines which cells in the linage will indeed switch. Our framework allows for investigating the role of Wor1 (and other regulators) in each of these steps, which will be the focus of future work. As a complement, our approach using *Saccharomyces cerevisiae* removes the context of the native system, allowing us to measure parameters of specific regulatory interactions directly.**

Major recommendations:

1. An interesting question would be to see if the current data can put a limit on how many generations the state that leads to later synchronous Wor1 activation can exist for. To put it the other way, how distantly related can two cells in a lineage be that both participate in synchronous Wor1 activation.

**We have added all of the pedigree examples for the Wor1-mGFP strain to the supplemental material (current Figure S7) and added an analysis of the number of divisions separating the establishment of the pedigree and cell switching.**

*Considering all of the examples of mixed-fate pedigrees observed for the Wor1-mGFP strain (Figure S7), there were between 1-4 cell divisions separating switching cells from the establishment of the mixed fate pedigree.*

2. A convenient way to confirm bistability arising due to the positive autoregulation of Wor1 in Saccharomyces would be to use the flow cytometry data used for Figure 5B. If one induces Wor1-GFP to a level that is around the half-maximal threshold, the expression level of mCherry driven from the Wor1 promoter at later generations should be heterogeneous i.e. some cells in the population should show strong mCherry expression and other should have none. An important control would be to show all cells have mCherry OFF in low Wor1-GFP expressed strain and all cells have mCherry ON in high Wor1-GFP expressed strain. This is an easy experiment to do with the strains reported in the paper

**In our *Saccharomyces cerevisiae* strains, the level of Wor1 protein is controlled by the level of exogenous hormone and not by the Wor1 regulatory sequences (which only drive the fluorescent reporter), hence there is no autoregulation in these experiments. By “opening” the Wor1 autoregulatory feedback loop, we were able to quantify its properties (current Figure 4). The half maximal threshold is actually defined by the level of Wor1 protein level that lead to a range of intermittent expression values of the mCherry reporter (see current Figure S10A). As the reviewer suggests, when the loop is “closed”, these intermittent values are likely unstable, either driving higher or lower levels of Wor1. It will be interesting to compare the dynamics of the closed loop however this will require more strain construction.**

Minor comments:

1. In Figure 1D, the authors can put Wor1-GFP in one panel and Wor1-mGFP in the other rather than two examples to show the differences in switching rate between the two constructs.

**In Figure 1D, we aim to show examples of individual switching events. The difference in switching rates between the Wor1-GFP and Wor1-mGFP constructs is a difference in the number of events we observe.**

2. Line 235, Page 215: Is the formation of polyploid cells associated with pseudohyphae formation. It might be interesting to some readers if the authors observe something similar.

**We only observed the formation of polyploid cells when the daughter cell was elongated. We believe this is similar to previous observations of abnormal nuclear division in elongated *Candida albicans* cells, which are cited in the text.**

3. Line 264, Page 216: The authors have mentioned here ‘they were always inherited across subsequent cell divisions’ but later they show that some of the cells in the lineage don’t turn on Wor1. The authors should revise this statement to avoid inconsistency.

**This line is in reference to high levels of Wor1 (similar to stable opaque cells). The progenitors of the mixed fate pedigrees do not express high levels of Wor1. The text was modified to clarify.**

*Once the expression of Wor1 in switching cells reached levels similar to stable opaque cells, they were always inherited across subsequent cell divisions indicating that the switch was complete.*

4. Line 252, Page 216: It is not exactly clear from the figure 3A-C if the time taken to each maximum level for Wor1 is 3 hours. In Figure 3C, the mother cells take ~6 hours to reach maximum expression levels. Therefore, it would be optimal to put a distribution of switching time and present the mean/standard deviation.

**The reference to 3 hours is the approximate time of increase in Wor1 levels from background fluorescence. The amount of time since the birth of the switching cell (6 hours for the example in former figure 3C) varies between cells. We clarify this observation in the text.**

*Consistent with the stochastic nature of switching, the starting time of different switching events did not depend on the time from the beginning of the experiment. Moreover, even the time between the birth of a switching cell and its switching varies from one cell to the next. For the set of examples shown in Figure 2D (n=21), maximum Wor1 activation is reached between 5.4 to 17.2 hours after cell birth (mean=9.9, standard deviation=3.2).*

5. In the figure 3B, the data can be plotted starting from the cell division rather than imaging time to show that they more or less take the same amount of time to reach activation thresholds. The mean and standard deviation of the time can be put on the graph. Additionally, the plots corresponding to mGFP and GFP need to be colored separately.

**As clarified in the previous comment the time to Wor1 activation from cell birth of the switching cell is not stereotypical. To emphasize the similarity in Wor1 activation between switching cells, we lined up the trajectories based on the approximate time cells reached maximum Wor1 levels (current Figure S5B). We also added distinction (using line type) between cells containing Wor1-GFP and Wor1-mGFP (current Figure 2D and Figure S5B).**

Reviewer #2:

In this manuscript, Ziv et al. investigate the stochasticity of Wor1 activation in Candida albicans which leads to rare switching from the white to opaque cell state. Wor1 is a master regulator of the opaque cell state. This is a Discovery Report and so the authors don't claim to have fully worked out the molecular basis of this stochasticity but instead report progress on this question by a combination of a few new techniques.  They argue that this environmental-induction free system is a useful model for understanding the basis of stochastic switching and the consequences of epigenetic changes to cell state.

The authors used single-cell microscopy with GFP labeled Wor1 to monitor Wor1 activation. Tracking cells (and their progeny) over time was a key part of their approach and so they used a microfluidic device with replicated cell growth chambers to enable them to follow individual cells over time while also having large enough numbers to be able to find rare stochastic switching events.  They find that dimerizing GFP increases the switching rate, and so they use this to increase the number of events they can observe while also validating their findings using a non-dimerizing GFP variant. They find that not only does Wor1 increase in expression in a subset of cells (as expected) but also that this state is inherited over two generations (they report data from a single granddaughter and two daughters).  Often, seemingly independent switching lineages in the same cell trap were actually related a few more generations back. However, not all lineages descended from that previous
generation switched, so while there is likely some protein or cell state that is inherited (supported by their Poisson analysis), it clearly does not surpass some threshold such that all descendants would be activated.  It isn't clear what this cell state is yet.

The authors construct a synthetic Wor1 positive autoregulatory reporter in S. cerevisiae and use it to estimate the Hill coefficient and the level of protein at half-max activation of the Wor1 promoter by Wor1. They find a big different in protein at half-max activation between the GFP-dimerized Wor1 and the non-dimerizing version.

They propose a two-step model for Wor1 activation with a potentiating phase where the cell state of some cells changes. These cells and their progeny are predisposed to switch, but only some end up doing so after another molecular event.

Overall, this seems a fairly clean paper. Microfluidics is a sensible technique to capture and track these events, particularly since they want to look at lineages of cells.  And their use of S. cerevisiae to study whether cooperativity might be involved is also reasonable.

**We thank the reviewer for the careful reading and accurate description of our paper.**

The pseudohyphal growth and mitosis part of the paper (204-240) felt much less satisfying than the rest of the paper. On the one hand, the association of pseudo-hyphae and GFP in the videos is unmistakable and so the authors obviously need to discuss it.  They claim (and show to some extent in figure 2A and also point to their videos) that pseudo-hyphae are neither necessary nor sufficient for switching.  It might be nice to given some percentages or a 2x2 table with the data on this. They also point out some unusual nuclear movement between mother and daughter during division.  Because this Discovery Report (as far as I understand) is partly meant to spur new research, it seems appropriate to leave this story unresolved. But it clearly merits follow-up - particularly as the pseudohyphal elongation seems to be transient.  A minor point on figure 2A - I wonder if a scatterplot of mother-daughter size might be more informative than separating them like they are. The white/opaque histograms could still be on the margins.

**We agree that the connection between filamentation and white-opaque switching is intriguing and requires additional work. We have added the percentages of switching cells that are more elongated then typical white cells to the text. We have also added the reviewers plot suggestion to Figure S2A.**

*For the strain containing the Wor1-GFP fusion protein, approximately 14% of mothers and 80% of daughters in pairs of switching cells were longer than the 95-percentile of white cell lengths (Figure 2A).*

On the basis of their cell traces over lineages, the authors claim that mothers and daughters increase Wor1 expression simultaneously (260). Fig 3C is meant to show this. But it seems to me that there is variation on the order of a few hours between these.  Is the order consistent across different sets of 4 cells? Is it related to size of the cells or concentration of GFP? They say that this supports the idea that the rate of switching is set early and continues apace even after cell division events (264). I wonder if there is anything more that can be done with this data to try to pin down when this second stochastic event happens relative to the first division of the mother.

**It is unclear what “variation in the order of a few hours” the reviewer is referring to as all four cells increase Wor1 expression at the same time (between 19-22 hours from the beginning of the experiment). Perhaps the reviewer is referencing the budding of the cells, which occur at two-hour intervals.**

In the discussion, on line 511 they propose that several cells switching at once may be adaptive.  This seems premature. It may be adaptive or it may not be.  There are a few thresholds in this model and some fractions of switching cells in a pedigree against a background of non-switchers and none of the evidence presented suggests that these have been fine tuned by selection.

**We agree that the adaptive value of coordinated switching is only a possibility and clarify this point in the text.**

*The occurrence of coordinated switching would be advantageous in situations where the rapid generation of the rare cell type could be beneficial. Although our experiments do not address the adaptive value of white-opaque switching, increasing the number of opaque cells could be valuable both for immune evasion or metabolic cooperation with white cells.*

Lines 524-535 present an interesting hypothesis tying the second stochastic step to translation efficiency and then points out that the Wor1 UTR reduces protein expression. I'd recommend putting this fact at the start of this paragraph since it is important context for the model that lines 524-533 lay out.

**We have reworded this section (current line 529).**

*According to this hypothesis, the first stochastic event during white-opaque switching might be a rare burst of low-level Wor1 transcription. This state would be heritable over only a small number of cell divisions, defining the small pedigrees of cells predisposed to switch.* *The second stochastic event would then create variation in the amount of Wor1 between cells, perhaps because of variation in translation efficiency, uneven partitioning during division or the stochastic expression of an inhibitor. Only certain cells in the pedigree would accumulate sufficient Wor1 protein to excite the Wor1 positive feedback loop, leading to high level Wor1 expression and subsequent cell-type switching.*

All in all, this paper describes a logical series of experiments that seems to fit the Discovery Report brief.  I have the suspicion that they can tease more out of their movies about the pseudohyphal connection, but that isn't essential and I may be wrong.

Minor point - figure 2C, the first measurements for the two daughters have very low GFP.  Is this real or is it some artifact of the image processing? If taken at face value, It suggests that GFP is very unevenly distributed between mother and daughter but the daughter GFP recovers one image later to be the same as the mother's.

**During the first time point of cell tracking, the cell is typically in the process of budding and tends to have lower than average autoflorescence.**


REVIEWER #2'S CROSS-COMMENTS:

Thanks for the opportunity to see the other review and comment. I think reviewer 1 caught several things that I missed. Their comments bring a few more ideas to mind.

Reviewer 1 mentions a scenario that involves Wor1 expression below a threshold for detection. I wonder if the authors could determine what this threshold would be. This made me go back to figure 3. There is an odd mismatch between the GFP levels of the no-GFP cells in panel A and the trajectories in panels B&C which appear to start consistently rising below this no-GFP level. It might be useful to show more trajectories like figure C, for both cells that will switch and cells that will not (like the grey circles in A). One difference between A and C is GFP vs. mGFP and so it would be good to know if the detection threshold somehow depends on which GFP variant.

**We have added a supplemental figure with more cell traces (current Figure S5A). Cells that are not actively switching have similar fluorescence levels to typical white cells that do not contain GFP.**

This question of detection thresholds also brings up an anomaly in Figure 5B. Why are the orange lines above the grey ones? Orange shouldn't have GFP.

**Inducing Wor1 in *S. cerevisiae* causes physiological effects that increased autofluorescence, this is now clarified in the text.**

*Inducing Wor1 or its GFP fusions also caused physiological effects such as cell clumping, leading to increased autofluorescence (in the Wor1 strains, orange lines in FIGURE 4B left) and bimodal induction distributions (in the Wor1-GFP strains, blue distributions in FIGURE S9).*

Reviewer 1 notes the large phylogenetic distance between C. albicans and S. cerevisiae in the engineering experiments. The argument is that the C. albicans transcription factor will act on the C. albicans regulatory sequence differently in S. cerevisiae than in C. albicans in such a way as to make the Hill coefficient estimates suspect. I don't think
phylogenetic distance per se is a problem. The question is whether there are other factors in C. albicans (or in S. cerevisiae) that are hindering (or helping) Wor1 activate its promoter. The control suggests that there aren't other factors in S. cerevisiae that are activating it on its own. So any effect would have to come through interaction with the Wor1 protein itself. But I think identifying any such factors if they exist would be beyond the scope of this paper. I think this experiment and the conclusions they draw are fine and probably better than if they had tried a similar sort of thing (e.g. measuring binding) using in vitro techniques. But the more careful phrasing and explanation of the inference and more explanation of how this is an advance over previous knowledge that Reviewer 1 asks for is warranted.

**We agree with the reviewer and modified the text.**

*We show here that Wor1 regulation of its own promoter is ultrasensitive even in the absence of other C. albicans regulators. While our measurements provide a baseline for the autoregulation of Wor1, other factors in C. albicans may change these parameters.*

Reviewer 1 presents a model for the two events that involves first a triggering event in a subset of cells that leads to increase in Wor1 levels and then a random failure of inhibition at some point right around the threshold that would let Wor1 activate itself. The authors didn't commit to any particular mechanism for these two events, and so I think they could add this possibility in their discussion. I wonder if candidates for this model could be identified - it would probably have to be a constitutive regulator where knockouts lead to increased opaque switching. There is probably data out there to narrow down to possible candidates, if any. I suppose it could also be induced in the first event along with Wor1. But all this is speculation.

**There are many intriguing possibilities about the underlying mechanisms responsible for the pattern of cell switching we observe. Future work will focus on elucidating the role of Wor1 and other known regulators in this process.**
